# *SOX11* contributes to the regulation of *GDF5* in joint maintenance

**DOI:** 10.1186/1471-213X-13-4

**Published:** 2013-01-29

**Authors:** Akinori Kan, Toshiyuki Ikeda, Atsushi Fukai, Takumi Nakagawa, Kozo Nakamura, Ung-il Chung, Hiroshi Kawaguchi, Clifford J Tabin

**Affiliations:** 1 Department of Genetics, Harvard Medical School, 77 Avenue Louis Pasteur, Boston, MA, 02115, USA; 2 Sensory & Motor System Medicine, Faculty of Medicine, University of Tokyo, Hongo 7-3-1, Bunkyo, Tokyo 113-8655, Japan; 3 Bone & Cartilage Regeneration, Faculty of Medicine, University of Tokyo, Hongo 7-3-1, Bunkyo-ku, Tokyo, 113-8655, Japan

**Keywords:** *SOX11*, *GDF5*, Joint maintenance, Articular cartilage

## Abstract

**Background:**

Individual skeletal elements of the vertebrate limbs arise through a segmentation process introducing joints in specific locations. However, the molecular pathways controlling joint formation and subsequent joint maintenance are largely unknown. In this study, we focused on *SOX11*, and its contribution to the regulation of *GDF5*, a secreted signal necessary for proper joint formation and postnatal joint homeostasis.

**Results:**

*Sox11* is initially expressed broadly in the murine cartilage condensations at early stages of skeletal development, but its expression is specifically increased in the forming joint interzone as is forms. *SOX11* overexpression can directly activate *GDF5* expression both in vitro and in micromass cell cultures prepared from chick limb buds. Conserved *SOX* family binding sites are present in the 5’ UTR region of the *GDF5* gene and we show *SOX11* can specifically bind to one of them. While misexpression of *Sox11* in developing chick limbs through RCAS virus infection does not induce *Gdf5* expression in ectopic locations, it does enhance its expression. To explore the roles of *Sox11* in joint homeostasis, we analyzed adult knee joints in an osteoarthritis mouse model where the medial meniscus and the medial collateral ligament were removed. We also analyzed knee joints from human subjects who underwent total knee replacement surgery. We find that *SOX11* is mainly expressed in the weight-bearing areas of knee joints, and its expression is decreased in degraded cartilage during progression of knee osteoarthritis in both mice and humans.

**Conclusions:**

This work implicates *SOX11* as a potential regulator of *GDF5* expression in joint maintenance and suggests a possible role in the pathogenesis of osteoarthritis.

## Background

Within the embryonic limb, skeletal development is initiated by aggregation of mesenchymal cells. These early condensations can subsequently adopt either of two distinct fates. They can give rise to cartilage and then bone through a process known as an endochondral ossification [1] or alternatively they can initiate a program leading to joint formation. In this latter process, a localized dense region known as an interzone is formed in the mesenchymal condensations, ultimately yielding a synovium, a capsule, ligaments and articular cartilage [2]. Although the molecular mechanisms regulating endochondral ossification have been fairly well-investigated [3,4], those controlling joint formation largely remain to be clarified. The subsequent mechanisms of joint maintenance are equally poorly understood.

Osteoarthritis (OA) is one of the most common diseases of the joint, which is characterized by cartilage destruction, synovial inflammation, and bone remodeling. Since articular cartilage has very poor regenerative capability, once the joint surface becomes degraded, it cannot be recovered. OA is a multifactorial disease which is influenced by mechanical, environmental and genetic factors [5,6].

*GDF5* is one of the genes that has been implicated in joint maintenance. *Gdf5* is expressed in the early developing joint interzone [7], and loss-of-function mutations in this gene cause abnormalities of a number of joints in mice [7,8]. However, misexpression of *Gdf5* does not induce ectopic joints, but rather causes an increase in chondrocyte differentiation and proliferation [9-11]. Interestingly, *GDF5* has also been reported to be required for joint integrity and homeostasis in humans. A single nucleotide polymorphism in the human *GDF5* promoter that reduces its transcriptional activity is associated with susceptibility to osteoarthritis [5]. Moreover, human loss-of-function mutations in the *GDF5* gene result in congenital skeletal disorders such as Hunter-Thompson and Grebe chondrodysplasia [12].

*SOX* genes are involved in cell type specification in a variety of tissues, including sex determination, neurogenesis and skeletal formation [13]. Of particular relevance, the chondrocyte lineage is established through the activity of *SOX9, SOX5* and *SOX6*[4,14]. *SOX9* binds to a regulatory element of the cartilage-specific type II collagen (*COL2A1*) gene [15], and its mutation in humans is known to cause a skeletal malformation called campomelic dysplasia [16,17]. Interestingly, the chondrogenic actions of *SOX* genes are also required for synovial joint morphogenesis [18], raising the possibility that *SOX* genes might similarly play integral roles in joint maintenance.

Based on analysis of their HMG domains, *SOX* genes can be separated into subgroups A-J [19]. Here, we examined the group-C *SOX* gene *SOX11*. We find that *Sox11* is expressed in the cartilage condensations in early stages of skeletal development in mice, but at later stages *Sox11* expression is notably increased in the joint interzone. We show that *SOX11* can activate *GDF5* expression and demonstrate that there is a direct binding site for *SOX11* in the 5’UTR of the *GDF5* gene in vitro. *Sox11* misexpression in chicks does not induce ectopic joint formation *in vivo*, but slightly enhances endogenous *Gdf5* expression. Finally, we find that SOX11 protein levels are decreased in degraded cartilage during osteoarthritic progression in humans and mice, indicating a possible role of *SOX11* in pathogenesis of osteoarthritis.

## Results

### The *Sox11* expression in developing limbs

To identify candidate genes that might play a role in regulating *GDF5* expression in developing joints, we conducted a preliminary screen of all the known *SOX* transcription factors. We examined the transcriptional activities of a reporter dependent upon the human *GDF5* promoter (-448/+319) following overexpression of each of the known human *SOX* genes in Hela and ATDC5 cells (Additional file 1). The genes that gave the most prominent results in this assay were the three group-C *SOX* genes (*SOX4, SOX11*, and *SOX12*) and of these, *SOX11* produced the strongest enhancement in both cell lines. The increased luciferase activitities induced by co-transfection of *SOX11* and the reporter construct containing the *GDF5* promoter were about 18 and 3.5 times higher (in the two cell lines respectively) than that of *SOX11* and the control reporter vector lacking the GDF5 promoter but containing the luciferase reporter (Additional file 1). We noted that *SOX11* activated transcription in the absence of the GDF5 promoter, albeit to a lesser degree, presumably due to non-specific interactions with the backbone of the reporter plasmid. This effect, which was not specific to *SOX11*, might be caused by the more stable α-helical structure of its transactivation domain than other *SOX* genes [20]. Nonetheless, *SOX11* had a clear and significant effect on the *GDF5* promoter above that seen with the vector alone. On the other hand, the overexpression of *SOX6 and 9,* which are known to be involved in cartilage differentiation in developing limb, had less effect on the activity of the *GDF5* promoter than that of *SOX11*. Based on this preliminary screen, we chose *SOX11* for further study.

**Figure 1:**
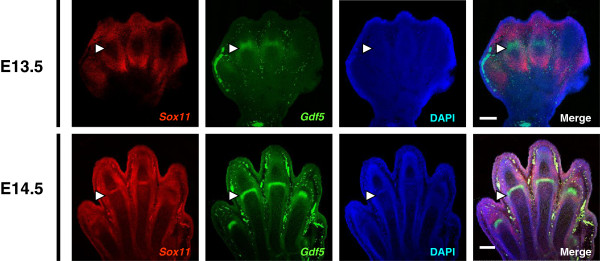
**Expression patterns of *Sox11 *and *Gdf5 *during joint formation in mouse embryos. **Double fluorescence section section in situ hybridization in hind limbs of mouse embryos (E13.5, 14.5). Confocal microscope images show (from left to right): *Sox11 *(red channel), *GDF5 *(green channel), DAPI staining of nuclei, and merged images. White arrowheads indicate the location of the interzone at prospective joint sites. Scale bars, 200 μm.

We first compared the expression pattern of *Sox11* and *Gdf5* by double fluorescence section in situ hybridization during an embryonic limb development of mice. At embryonic stage 13.5 (E13.5), when joint interzones are just starting to form between the metatarsals and tarsal elements, *Sox11* is expressed in the cartilage condensations, showing a distinct expression pattern from the joint-specific *Gdf5* (Figure 1). However, at E14.5, the level of *Sox11* transcription is specifically increased at the interzone in the prospective joint region and co-localized with *Gdf5* (Figure 1).

### Increase of endogenous *GDF5* expression by *SOX11* overexpression

**Figure 2:**
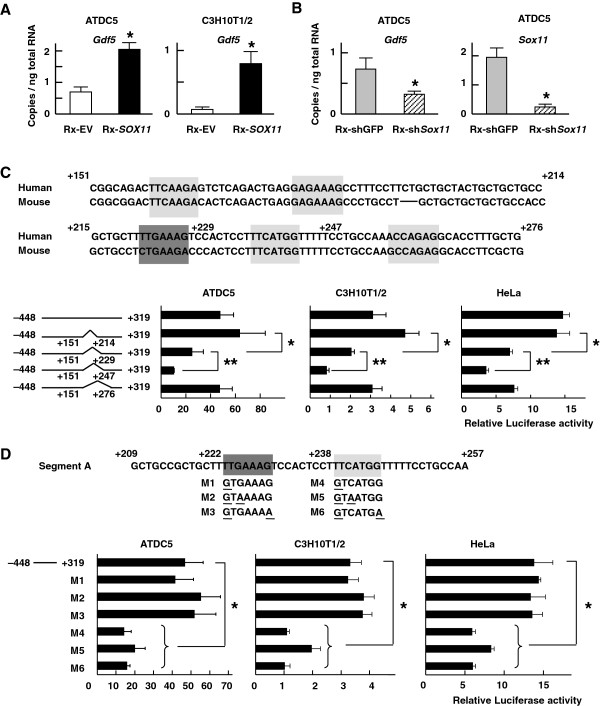
**Determination of a core responsive element of *SOX11 *in the *GDF5 *promoter. **(**A**) Levels of *Gdf5 *mRNA in ATDC5 and C3H10T1/2 cells retrovirally transfected with the control vector (Rx-EV) or the vector expressing human *SOX11 *(Rx-*SOX11*). mRNA levels were determined by quantitative RT-PCR, normalized to *Gapdh*, and expressed as means (bars) ± SDs (error bars) for 4 wells/construct. *P < 0.05 vs. Rx-EV. (**B**) *Gdf5 *and *Sox11 *mRNA levels in ATDC5 cells retrovirally transfected with the shRNA vector targeting GFP (Rx-shGFP) or targeting mouse *Sox11 *(Rx-sh*Sox11*). *P < 0.05 vs. Rx-shGFP. (**C**) Deletion analysis using constructs lacking putative *SOX*-family-binding motifs in the proximal human *GDF5 *promoter (+151/+276) in ATDC5, C3H10T1/2, and HeLa cells. Five putative SOX family binding sites are shown (+159 to +165, +179 to +185, +222 to +228, +238 to +244, +258 to +264). The site with 1 base difference from the ideal binding site is shaded in dark gray, and those with 2 bases difference are indicated in light gray. Luciferase assays were carried out as above, in ATDC5, C3H10T1/2 and HeLa cells. *P < 0.05 vs. deletion of +151/+214 **P < 0.05 vs. deletion of +151/+229. (**D**) Identification of a core responsive element of *SOX11 *in the *GDF5 *promoter (segment A: +209/+257). Site-directed mutagenesis analysis with 6 mutated constructs (M1–M6). Base changes for each mutation as shown. Luciferase assays were carried out as above, in ATDC5, C3H10T1/2 and HeLa cells. The data for the luciferase assay are expressed as means (bars) ± SDs (error bars) of Relative luciferase activity for 4 assays/construct. *P < 0.05 vs. wild type promoter, –448/+319.

To examine the capacity of *SOX11* to regulate cellular *GDF5* expression, we created variants of the murine chondrogenic ATDC5 and fibroblastic C3H10T1/2 cell lines in which we stably introduced human *SOX11* through retroviral infection. The resultant *SOX11* overexpression increased endogenous mRNA levels of *Gdf5* in both cell lines (Figure 2A). Conversely, the *Gdf5* mRNA level was suppressed (to a level 44% of that seen in controls) by the retrovirally mediated shRNA knock-down of endogenous *Sox11* activity (to a level 12% of that seen in controls) in ATDC5 cells (Figure 2B).

*SOX11* has three conserved domains: an N-terminus SRY-related HMG domain (amino acids 1–125), an acid-rich domain (amino acids 218–250) and a C-terminus domain (amino acids 386–441) (Additional file 2), which contains potential transcriptional regulatory regions [21]. To determine the domains that are required for *GDF5* expression, we created constructs lacking each domain, called *SOX11*ΔN (lacks amino acid 1–134), *SOX11*ΔAR (lacks amino acid 200–309) and *SOX11*ΔC (lacks amino acid 297–441), respectively, and examined their ability to transcriptionally regulate the *GDF5* promoter. The lack of the N-terminus HMG domain or C-terminus domain abrogated the transcriptional response induced by full-length *SOX11* overexpression (Additional file 3A). Moreover, the endogenous *Gdf5* mRNA level was significantly suppressed by constructs carrying deletions in either of these domains in ATDC5 cells by retroviral infection (Additional file 3B). In contrast, the construct lacking the acid-rich domain did not abrogate *Gdf5* expression (Additional file 3B), indicating that the N-terminus and C-terminus domains, but not the acid-rich domain, play important roles in mediating *SOX11* function with respect to *GDF5* expression.

### Identification of a core *SOX11*-responsive element in the *GDF5* promoter

We found five clustered sequences similar to the putative *SOX* family consensus binding site (A/T)(A/T)CAA(A/T)G in this region (Figure 2C), that were highly conserved between humans and mice [22]. One of them was one-base different from the published consensus binding site (shaded in dark gray), and the others were two-bases different (shaded in light gray) (Figure 2C). We created four reporter constructs deleting these motifs: deletion of first two binding sites (+151 bp and +214 bp), first three binding sites (+151 bp and +229bp), first four binding sites (+151 bp and +247 bp), or all five binding sites (+151 bp and +276 bp); and compared their transcriptional activities in a dual-luciferase assay. We observed a stepdown in the luciferase activity induced when a deletion is introduced between +215 bp and +247 bp, which contains two adjacent *SOX* family binding motifs. These were thus candidates for *SOX11* binding sites in the *GDF5* promoter. A 49 bp element (+209 bp/+257 bp), containing the identified putative binding sites, was defined as segment A (Figure 2D).

To further determine the core responsive sequence in segment A, we created mutations in the identified motifs (mutation 1 at +222 bp, mutation 2 at both +222 bp and +224 bp, mutation 3 at both +222 bp and +228 bp, mutation 4 at +238 bp, mutation 5 at both +238 bp and +240 bp, and mutation 6 at both +238 bp and +244 bp). Transactivation by *SOX11* was decreased only by mutations 4,5 and 6, not by mutations 1,2 or 3, and the inhibitory effect among mutations 4, 5 and 6 were comparable, indicating that the thymine at the +238 bp nucleotide is crucial for the binding between *GDF5* promoter and *SOX11* (Figure 2D).

Previous reports suggest that a single nucleotide polymorphism (SNP) rs143383 (+45 bp) in the 5’UTR region is related to *GDF5* expression and its postnatal joint maintenance [5]. We found a putative *SOX* family binding motif at (+57/+63) just downstream of the SNP. We examined the potential ability of *SOX11* to activate gene transcription through this motif in a luciferase assay. Deletion of the *SOX* motif near the SNP did decrease transcriptional activity induced by *SOX11* overexpression. However, the one nucleotide mutation at the +238 bp (identified above) suppressed it more extensively, indicating the binding motif at +238 has a stronger affinity for the *GDF5* promoter (Additional file 4).

**Figure 3:**
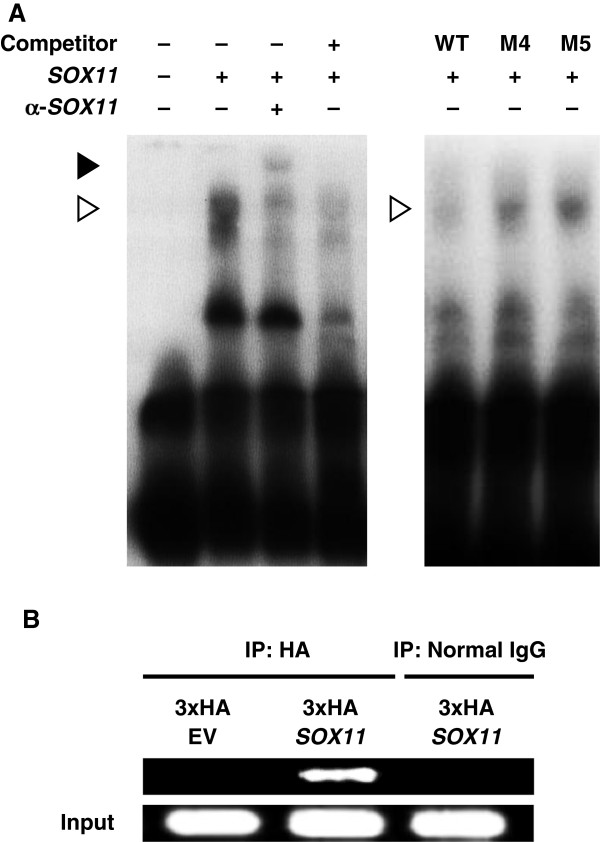
**Direct binding between *SOX11 *protein and *GDF5 *promoter. **(**A**) Electro mobility shift assay (EMSA) for binding of the wild-type (WT) and mutated probes with human SOX11 protein. An open arrowhead indicates the shifted bands of the SOX11-DNA probe complex, and a solid arrowhead indicates the band super shifted by an antibody to SOX11. Cold competitions with a 50-fold excess of the unlabeled WT probe or the mutated probes are shown. Detailed information about each mutated nucleotide is indicated in Figure 2D. (**B**) Chromatin immunoprecipitation (ChIP) assay for *in vivo *binding of SOX11 and the segment A. Cell lysates of HeLa cells transfected with empty vector (3xHA EV) or *SOX11* (3xHA *SOX11*) were amplified with a primer set (+98/+364) spanning segment A.

We further examined the binding of *SOX11* to segment A in electromobility shift (EMSA) and chromatin immunoprecipitation (ChIP) assays. In EMSA, an oligonucleotide probe detected a complex formation between a segment A and in-vitro translated SOX11 protein, which was supershifted by an antibody to SOX11, indicating the direct binding of SOX11 to the segment A (Figure 3A). Moreover, cold competition with an excess amount of the unlabeled wild-type probe reduced the complex formation. Specificity of SOX11 antibody was confirmed by the absence of a supershift band with a non-immune normal IgG (Additional file 5). A chromatin immunoprecipitation (ChIP) assay was performed using HeLa cells transfected with *SOX11* linked to three tandem repeats of the HA tag. Pull down with an antibody to the HA tag showed *in vivo* binding of SOX11 to the *GDF5* regulatory region including segment A (Figure 3B). Specificity was confirmed, as SOX11 was not immunoprecipitated by the overexpression of the empty vector or by a control non-immune IgG antibody.

### Involvement of *SOX11* in cartilage formation in vitro

**Figure 4:**
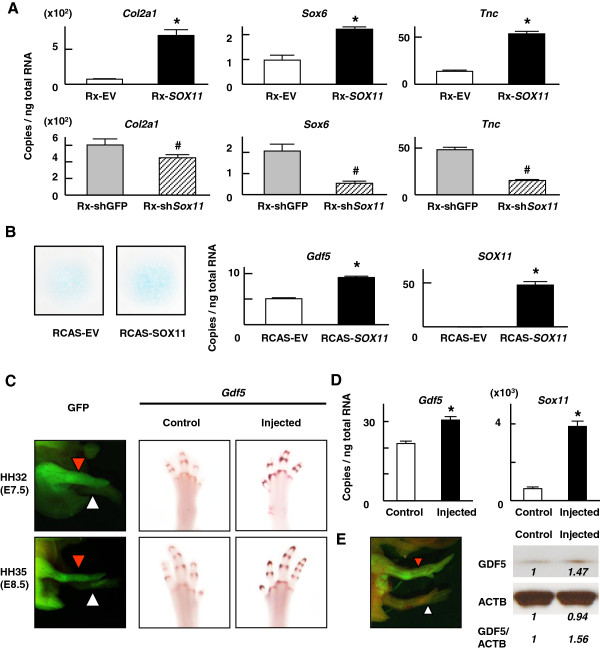
**Contribution of *Sox11 *to *Gdf5 *expression in developing limbs. **(**A**) mRNA levels of *Col2a1*, *Sox*6 and *TenasinC (Tnc) *in ATDC5 cells that were retrovirally transfected with a control vector (Rx-EV) or human *SOX11 *(Rx-*SOX11*) and the short hairpin RNA directed against GFP (Rx-shGFP) or against mouse *Sox11* (Rx-sh*Sox11*). mRNA levels were determined by quantitative RT-PCR and expressed as means (bars) ± SDs (error bars) for 4 wells/construct. *P < 0.05 vs. Rx-EV. ^#^P < 0.05 vs. Rx-shGFP. (**B**) Alcian blue staining of micromass cell cultures and mRNA levels of *Gdf5* and *SOX11 *in those cultures after infection of RCAS virus encoding human *SOX11*. *P < 0.05 vs. RCAS-EV. (**C**) Whole mount in situ hybridization for *Sox11 *and *Gdf5 *in wild type chick embryos at HH stage 32 or 35 and in embryos after injection of RCAS virus encoding GFP-fused chick *Sox11 *at HH stage 10. The GFP fluorescence after the injection is also shown. Red arrows indicate the injected right hind limbs, while white arrows indicate the uninjected left hind limbs for control. Scale bars, 1 mm. (**D**) mRNA levels of chick *Sox11*, and *Gdf5 *genes of hind limbs at HH stage 35 (E8.5) after the RCAS injection of GFP-fused chick *Sox11*. The uninjected left hind limb is used as control. *P < 0.05 vs. control. (**E**) Western blotting for *Gdf5 *and β-Actin (ACTB) in HH stage 35 (E8.5) chick hind limbs after the RCAS injection of GFP-fused chick *Sox11*. The uninjected left hind limb is used as control. The GFP fluorescence in the embryos used for Western blotting analysis is also shown. Red arrow indicates the injected hind limb and white arrow shows the control hind limb. Densitometric analysis was performed with Image J software.

*GDF5* is known to have chondrogenic ability in vitro and *in vivo*[9-11]. Therefore, we next examined the potential ability of *SOX11* to stimulate chondrogenic differentiation in vitro. The endogenous mRNA levels of chondrogenic marker genes, *Col2a1, Sox6* and *TenascinC*[23] were all increased by the retroviral introduction of human *SOX11* into ATDC5 cells (Figure 4A). Moreover, a short hairpin RNA (shRNA) directed against the endogenous mouse *Sox11* decreased the expression of the three chondrogenic marker genes (Figure 4A). A similar chondrogenic effect of *SOX11* was observed in micromass cell cultures of chick limb bud cells after infection of an RCAS virus encoding human *SOX11*, as indicated by an increase in Alcian blue staining (Figure 4B). The endogenous *Gdf5* mRNA level was concomitantly increased in the micromass cultures by the infection of RCAS virus encoding *SOX11* (Figure 4B).

### Misexpression of *Sox11* in chicks enhances *Gdf5* expressions *in vivo*

To know the effect of *Sox11* overexpression *in vivo*, we injected RCAS virus encoding GFP fused to the chick *Sox11* cDNA into right chick lateral plate mesoderm at HH stage 10 (day 1.5), and harvested the limb at HH stage 32 or 35 (day 7.5 or 8.5, respectively). After confirming GFP fluorescence (Figure 4C), we performed whole mount in situ hybridization on the injected limbs with a riboprobe for chick *Gdf5*. We used the non-infected contralateral side of the same embryo as a control. Misexpression of *Sox11* did not induce additional domains of *Gdf5* expression, indicating *Sox11* is not sufficient to drive its expression in ectopic locations (Figure 4C). However, when we harvested the hind limbs and examined the expression level of G*df5*, we saw a slight but significant enhancement following *Sox11* overexpression (Figure 4D). The increased expression of GDF5 protein was also confirmed by Western blotting (Figure 4E). These results suggest that *Sox11* can at least partially contribute to the regulation of *Gdf5* expression but it is more than likely that there are other molecules involved in its expression during embryonic development.

### Decrease of *SOX11* expression during cartilage degradation

**Figure 5:**
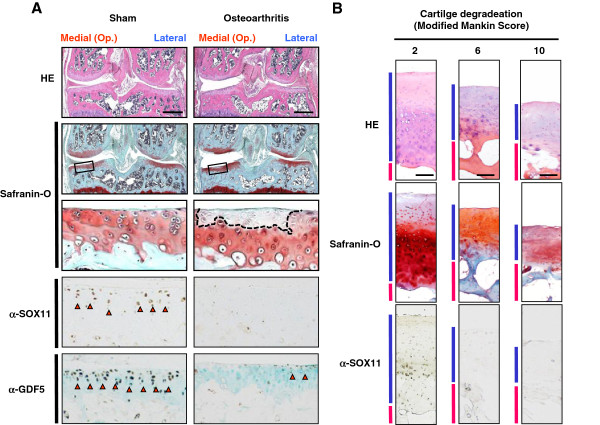
**Contribution of *SOX11 *to articular cartilage homeostasis in mice and humans. **(**A**) Histological findings of articular cartilage after creating an experimental osteoarthritis model that induces instability in the knee joints of 8-week-old mice. HE and Safranin-O stainings, as well as immunohistochemical staining with an antibody to SOX11 (α-SOX11) or GDF5 (α-GDF5) were performed 4 weeks after surgery. A sham operation was performed using the same approach. Operations were performed in the medial side of knee joints. Dotted line highlights the region where Safranin-O staining is lost in the arthritic model. Red arrowheads indicate positive signal for SOX11 or GDF5. Scale bars, 500 μm. (**B**) H&E staining, Safranin-O staining and immunostaining with an antibody to human SOX11 in human knee cartilages, obtained as surgical specimens of total knee arthroplasty. Cartilage degradation was evaluated by the modified Mankin scoring system. The blue and red side bars indicate the articular cartilage layer and subchondral bone layer, respectively. Scale bars, 500μm.

To know the role of *SOX11* in cartilage homeostasis, we next created an experimental osteoarthritis (OA) model through surgical induction of instability in the knee joints of 8-week-old mice [24]. *Sox11* is normally expressed at a high level in the weight-bearing regions of the mature joint, although it is less expressed in the peripheral weight bearing zone or non-weight bearing zone such as edge of femoral condyle, tibial plateau or intercondylar space (Additional file 6). Four weeks after surgery, the medial knee joints exhibited cartilage degradation, as demonstrated by a decrease in Safranin-O staining (Figure 5). Immunohistochemical analysis revealed that both *Sox11 and Gdf5* expression levels were decreased in the degraded cartilage in the medial knee joint, whereas they were still strongly expressed in the intact cartilage from the sham-operated knees or from the lateral knee joint of OA-operated knees (Figure 5A) (Additional file 7). These results were consistent among multiple sections obtained from the center of the weight-bearing area in different mouse knee joints (n = 6/6 slides) (Figure 5A).

Finally we analyzed 10 samples of human knee joints that had undergone total knee arthroplasty. Immunohistochemical analysis revealed that *SOX11* expression was decreased from early stages of osteoarthritis progression, as assessed by decreased Safranin-O staining and modified Mankin’s cartilage degradation scores (Figure 5B) (Additional file 8). Specificity of *SOX11* antibody was confirmed by using the antibody of non-immune IgG (Additional file 9).

## Discussion

In the present study, we identify *SOX11* as a molecule contributing to the regulation of *GDF5* expression during joint formation and maintenance. In previous studies, *SOX11* was identified as an essential molecule for neuronal maturation, however its molecular function in developing limbs had not been explored [25,26]. We found that *SOX11* is expressed in the skeletal elements of the developing limbs and that its expression is increased in joint tissue as it develops (Figure 1). *Sox11* deficient mice show a range of skeletal morphological abnormalities [27]. However, no defects were observed in the program of joint formation per se, and the resulting limb phenotype is not severe [27]. It is more than likely that many genes are involved in joint formation and their function may be redundant.

We show that *SOX11* can enhance *GDF5* expression in vitro (Figure 2). *SOX* proteins are known to bind the 5’ UTR of target genes to modulate their transcriptional activities. [28,29] We find that, indeed, *SOX11* can bind the 5’UTR of the *GDF5* gene. Although *SOX11* enhanced chondrogenic proliferation in vitro, its misexpression *in vivo* did not affect the gross morphology of limb nor the mRNA expression patterns of *Gdf5* (Figure 4C). However, *SOX11* overexpression increased the mRNA levels of *Gdf5* both in micromass cell culture from chick limb bud cells and in misexpression experiments *in vivo*, albeit to a small extent (Figure 4). While *SOX11* is not requisite for joint formation, our results suggest that *SOX11* is likely to at least partially contribute to the regulation of *GDF5* expression during that process.

Finally, to investigate the roles of *SOX11* in joint homeostasis, we examined its expression pattern in an osteoarthritis experimental model of knee joints in adult mice and human samples after total knee replacement surgery (Figure 5). A single nucleotide polymorphism in the *GDF5* gene has been reported to be related to susceptibility to osteoarthritis [5,30]. Although the *SOX11* binding site in our study seems to be independent of this reported SNP, reverse phase protein arrays in osteoarthritic and normal cartilage tissue identified *SOX11* as one of the molecules down-regulated in osteoarthritic cartilage [31]. Consistent with these results, *SOX11* expression was decreased in degraded cartilage both in mouse experimental osteoarthritis model and in human samples of osteoarthritis. These data support the speculation that *SOX11* could be important not only for embryonic joint formation, but also for joint homeostasis in articular cartilage in adults. This could be tested by assessing whether *Sox11* deficient mice have increased incidence of developing OA relative to their wild type littermates.

Joint formation is a complex and multistep process. Another molecule essential for joint formation is *Wnt9a*[32]. *Wnt* signaling inhibits chondrogenesis and promotes joint formation [32,33]. Several joints are abnormally formed in the *Wnt9a*-deficient mice [34]. The process of joint formation likely includes local induction of β-catenin/*Wnt9a* activity, which serves to block chondrogenesis and to subsequently maintain the interzone space. In parallel, signals secreted from the interzone, potentially including *Gdf5*, act on neighboring cartilage elements to prevent induction of a new interzone in the vicinity. Cells taken from prospective autopod joint sites using a microsurgical procedure displayed a propensity to undergo chondrogenesis following treatment with exogenous recombinant *Gdf5*[35], indicating that the formation of the mature articular cartilage is induced by the stimulation of chondrogenesis by secreted proteins such as *Gdf5*. We suggest that *SOX11* could be one of the molecules involved in the later stages of joint development, contributing to the maturation of articular cartilage, at least in part through enhancing *GDF5* expression.

## Conclusions

In this study, we have identified *SOX11* as being capable of directly regulating *GDF5* in vitro and showed its partial contribution to the *in vivo* regulation of *GDF5* expression during joint formation and homeostasis. The further identification of molecules acting upstream of *GDF5* will not only provide further understanding of joint formation, but will also potentially lead to insight into the pathogenesis of human joint diseases such as osteoarthritis.

## Methods

### Animals

All animal experiments were performed according to a protocol approved by the Institutional Animal Care and Use Committee (IACUC) at Harvard Medical School and the Animal Care and Use Committee of the University of Tokyo.

### Cell cultures

DF-1 cells were obtained from the American Type Culture Collection (ATCC), and ATDC5, C3H10T1/2, and HeLa cells were obtained from the RIKEN BioResource Center Cell Bank (Ibaraki, Japan). DF-1, C3H10T1/2 and HeLa cells were maintained in Dulbecco’s modified Eagle’s medium (DMEM) containing 10% FBS (Invitrogen, Carlsbad, CA), supplemented with L-glutamine (2 mM), penicillin (100 U/mL), and streptomycin (100 mg/mL). ATDC5 cells were maintained in DMEM/Ham’s F-12 (1:1) containing 5% FBS, human transferrin (10 μg/mL), sodium selenite (30 nM) and the above supplements.

### Plasmid construction

The human *SOX11* gene was PCR-amplified, and cloned into pCMV-HA plasmid (Clontech, Mountain View, CA). The *SOX11* cDNA was then excised from pCMV-HA and cloned into pMx-IRES-Bsr retroviral expression plasmid [36] and pCMV-3x HA tag vector, which was modified from the original pCMV-3Tag-1A vector (Stratagene, La Jolla, CA). Human *GDF5* cDNA was cloned into the pMx-IRES-Bsr. Chick *Sox11* cDNA was cloned into the pCAG-GFP vector [37], and chick cDNA with GFP fragment was excised and cloned into RCAS-BP(A) vector [38]. The deleted mutants of the functional domains in *SOX11* were created by PCR. For gene silencing, short hairpin RNA (shRNA) sequences directed against the mouse *Sox11* gene and GFP for control were designed by BLOCK-iT RNAi Designer (Invitrogen) and their expression vectors were constructed with piGENEmU6 vector (iGENE Therapeutics, Tokyo). The U6 promoter and the insert were excised from the piGENEmU6 and cloned into the pMx-IRES-Bsr. The 5’-flanking sequence (-448 bp/+319 bp) of the human *GDF5* promoter was amplified by PCR and cloned into pGL3-basic plasmid (Promega, Madison, WI). Mutated constructs were created by PCR. Sequences of all the plasmids were confirmed by an ABI PRISM 3100-Avant Genetic Analyzer (Applied Biosystems, Foster City, CA). Primers for plasmid construction and shRNA design are shown in Additional file 10.

### Luciferase assay

Transfection of ATDC5, C3H10T1/2, or HeLa cells was performed in duplicate in 24-well plates using FuGene 6 with plasmid DNA (200 ng of pGL3 reporter vector, 100 ng of effector vector and 4 ng of pRL-TK vector (Promega) for internal control per well). Cells were harvested 48 hours after transfection. Luciferase assays were performed with a dual luciferase reporter assay system (Promega) using the LMax luminometer (Molecular Devices, Sunnyvale, CA). Results were shown as the ratio of firefly activity to Renilla activity. Four wells were transfected with each construct (2 wells for Figure S1) and experiments were repeated at least twice in each cell line for each construct.

### Retroviral gene transfer

Two x 10^6^ Plat-E cells were plated in 60-mm dishes, and were transfected with 3 μg pMx vector using Fugene (Roche) on the following day. Forty-eight hours after transfection, the medium was collected and used as the retrovirus supernatant, which was applied to the ATDC5 or C3H10T1/2 cells. Selection of the retrovirally introduced ATDC5 or C3H10T1/2 cells was started after 2 days in the medium containing 10 μg/ml of blasticidin. After 2 weeks, the surviving cells were collected and assayed.

### Quantitative RT-PCR analysis

Total RNAs from cells were isolated with an RNeasy Mini Kit (Qiagen) and each sample was treated by DNase, reverse-transcribed with a QuantiTect reverse transcription kit (Qiagen), and used as a template for the 2nd step PCR. Full-velocity SYBR Green PCR Master Mix (Stratagene, La Jolla, CA) was used for the 2nd step PCR and fluorescence detection was performed with MX3000P (Stratagene). Copy numbers of target genes’ mRNA were quantified by a dilution series of standard templates and adjusted with human, rodent or chick *Gapdh* as an internal control. Four pipetting replicates were performed for each reaction. Primers for quatitative RT-PCR are shown in Additional file 10.

### Electrophoretic mobility shift assay (EMSA)

SOX11 protein was in-vitro translated using the TNT Quick coupled transcription/translation system (Promega). EMSA was carried out using a DIG Gel Shift kit (Roche), according to manufacturer’s instructions. Binding reactions were incubated for 30 minutes at room temperature. For competition analyses, a 50-fold excess of unlabeled competitor probe was included in the binding reaction. For the super shift experiments, 200ng of anti-SOX11 antibody (Santa Cruz Biotechnology, Santa Cruz, CA) was added after 30 minutes of binding reaction, and the reaction was incubated for an additional 60 minutes at room temperature. Samples were loaded onto Novex 6% Tris–borate–EDTA gels (Invitrogen) and electrophoresed at 120V for 60 minutes.

### Chromatin immunoprecipitation (ChIP) assay

A ChIP assay was performed with an OneDay ChIP kit, according to manufacturer’s instructions (Diagenode, Liege, Belgium). HeLa cells were transfected with 3x HA tagged empty vector or the *SOX11* vector using FuGene 6 as described above. *In vivo* crosslinking was performed 72 hours after transfection. To shear genomic DNA, the lysates were then sonicated on ice ten times for 30 seconds each. For immunoprecipitation, 1μg of anti-HA antibody (Abcam, Cambridge, MA) or non-immune normal rabbit IgG were used.

### Histological analyses of mouse embryos

For section in situ hybridization, partial coding sequences of mouse *Sox11* and *Gdf5* gene with the T7 promoter sequence were obtained by PCR amplification. A labeled antisense riboprobe for *Sox11* was prepared with T7 RNA polymerase using a DIG RNA labeling mix (Roche), and that for *Gdf5* was prepared using a Fluorescein RNA labeling mix (Roche). The C57BL/6 mouse embryos (E13.5-E14.5) were prepared as previously described [39]. The *Sox11* probe was detected using an anti-DIG antibody-coupled POD and TSA plus cyanine 3 system (PerkinElmer, Waltham, MA). The *Gdf5* probe was detected using an anti-Fluorescein antibody-coupled POD and TSA plus Fluorescein system (PerkinElmer). Images were obtained using an SP2 inverted confocal microscope (Leica, Wetzlar, Germany). For immunohistochemistry, tissues were fixed in 4% paraformaldehyde, embedded in paraffin. The sections were incubated with primary antibodies to SOX11 (Sigma; 1:200) or GDF5 (Abcam; 1:100). Immunodetection was performed using CSA II (DakoCytomation, Carpinteria, CA).

### Misexpression experiment in chick embryos

A chicken fibroblast cell line DF-1 was plated in a 60 mm dish the day before transfection. Three μg of RCAS BP (A) plasmids were introduced with Fugene6 transfection regent (Roche). The cells were cultured for 3 weeks, and the supernatant was collected and used as a virus stock. Preparation of micromass cell culture and the infection of RCAS virus were performed as previously described [32]. The RCAS viruses were injected into the lateral plate mesoderm of Hamburger Hamilton (HH) stage 10 chick embryos (day 1.5) to target gene misexpression to developing limbs as previously reported [38,40]. Embryos were harvested at HH stage 32 or 35 (or day 7.5, 8.5, respectively). Tissue preparations for whole mount in situ hybridization were performed as described above. Digoxigenin (DIG)-labeled antisense riboprobes were detected using anti-DIG antibody-coupled AP and NBT/BCIP solution (Roche). For quatitative RT-PCR analysis, total RNAs from chick limbs at HH stage 35 were isolated with an RNeasy Mini Kit. For Western blotting, hind limbs of HH stage 35 were homogenized with TissueLyser (Qiagen). Anti-GDF5 antibody (Abcam; 1:1000) and Anti-β-Actin antibody (Abcam; 1:1000) were used for primary antibodies and Goat anti-Rabbit antibody (Jackson ImmunoResearch; 1:10000) was used for a secondary antibody. Densitometry analysis was performed with ImageJ software.

### Osteoarthritis experiment

The surgical procedure to create an osteoarthritis experimental model of knee joints was carried out as previously reported [24], transecting the medial collateral ligament and medial meniscus of 8-week-old mice. A sham operation was performed on the contralateral knee. Four weeks after surgery, the entire knee joints were dissected, decalcified, and underwent hematoxylin-eosin (HE) and Safranin-O-fast green stainings, and/or immunohistochemical staining with the antibody to SOX11 and GDF5.

### Human samples

We obtained human samples from individuals undergoing total knee arthroplasty after obtaining written informed consent as approved by the Ethics Committee of the University of Tokyo. We histologically assessed cartilage degradation according to the modified Mankin scoring system. [41,42].

### Statistical analysis

A Mann-Whitney test was performed for the statistical analyses of luciferase assays and quantitative RT-PCR. p < 0.05 was deemed significant.

## Competing interests

The authors declare that they have no competing interests.

## Authors’ contributions

AK carried out the experiments described in this report. TI, KN and UC participated in the design of the study. AF created the experimental model of osteoarthritis in mice and TN collected human samples. AK, CT and HK analyzed the data. AK and CT wrote the manuscript. All authors read and approved the final manuscript.

## Supplementary Material

Additional file 1: Figure S1 Screening for candidate *SOX *genes activating *GDF5 *promoter. Luciferase assays for the transcriptional activity of the *GDF5 *promoter by the overexpression of different *SOX *family molecules in ATDC5 and HeLa cells transfected with *GDF5 *promoter region (-448/+319) ligated to the luciferase-reporter gene. Data are expressed as means (bars) ± SDs (error bars) of Relative luciferase activity for two assays/construct. EV: empty vector, the vector containing the luciferase transgene but lacking the GDF5 promoter. Click here for file

Additional file 2: Figure S2 Conserved domains in the SOX11 protein sequence among humans, mice and chicks. Amino acid sequences coding SOX11 protein is shown and the conserved domains among species are indicated by black boxes. Click here for file

Additional file 3: Figure S3 Identification of functional *SOX11 *domains which are required for *GDF5* expression. (A) Luciferase assays for the transcriptional activity of the reporter driven by the *GDF5 *(-448/+319) promoter, in the context of co-transfection with deletion mutants that lack N-terminus domain (Rx-ΔN), Acid-rich domain (Rx-ΔAR), or C-terminus domain (Rx-ΔC) in ATDC5, C3H10T1/2 and HeLa cells. Data are expressed as means (bars) ± SDs (error bars) of Relative Luciferase activity for four assays/construct. (B) Endogenous *Gdf5 *mRNA expressions in ATDC5 cells retrovirally transfected with the deletion mutants of *SOX11*. mRNA levels were determined by quantitative RT-PCR and expressed as means (bars) ± SDs (error bars) for 4 wells/construct. Click here for file

Additional file 4: Figure S4 Luciferase assay for deletion of *SOX *family binding site near the rs143383. Data are expressed as means (bars) ± SDs (error bars) of Relative Luciferase activity for four assays/construct. *P < 0.05 vs.–448/+319 promoter. **P < 0.05 vs.–448/+319 promoter lacking the +51/+75 region. Click here for file

Additional file 5: Figure S5 Electromobility shift assay (EMSA) for specific binding of the GDF5 promoter with human SOX11 protein. The wild-type probe for segment A was used. (Detailed information is shown in Figure 2). An open arrowhead indicates the shifted bands of the SOX11-DNA probe complex, and a solid arrowhead indicates the band supershifted by an antibody to SOX11. The supershift band is absent by a non-immune normal IgG. Click here for file

Additional file 6: Figure S6 Immunostaining with an antibody to SOX11 in multiple regions of mouse knee joints. Red arrowheads indicate positive signal for SOX11, while white arrowheads showed scarcity of signal for SOX11. Immunostaining was detected by DAB, which was followed by counterstaining with methyl green. mm: medial meniscus, lm: lateral meniscus, ACL: anterior cruciate ligament. Click here for file

Additional file 7: Figure S7 Immunostaining in the lateral compartment of knee joints in osteoarthritis model of mice. Immunostainings for SOX11 or GDF5 were detected by DAB, which was followed by counterstaining with methyl green. Red arrowheads showed positive signal for SOX11 or GDF5. Click here for file

Additional file 8: Figure S8 Decrease of the SOX11 expression during osteoarthritis progression. Safranin-O staining and immunostaining, with an antibody to SOX11, of human knee cartilages. Other representative samples are shown in Figure 5B. Cartilage degradation was evaluated by the modified Mankin scoring system. Red arrowheads indicate positive signal for SOX11. Scale bars, 500μm. Click here for file

Additional file 9: Figure S9 Immunostaining, with an antibody to SOX11 or non-immune normal IgG, of human cartilages. Immunostaining was performed on human knee cartilage with a modified Mankin Score = 2. Click here for file

Additional file 10: Table 1 Primer sequences for plasmid construction, shRNA or RT-PCR analysis used in this study. Click here for file
