# Supplementary figures and images for: SOX11 contributes to the regulation of GDF5 in joint maintenance
Source: BMC Dev Biol. 2013 Jan 29;13:4. doi: 10.1186/1471-213X-13-4 (PMC3760452; doi:10.1186/1471-213X-13-4)

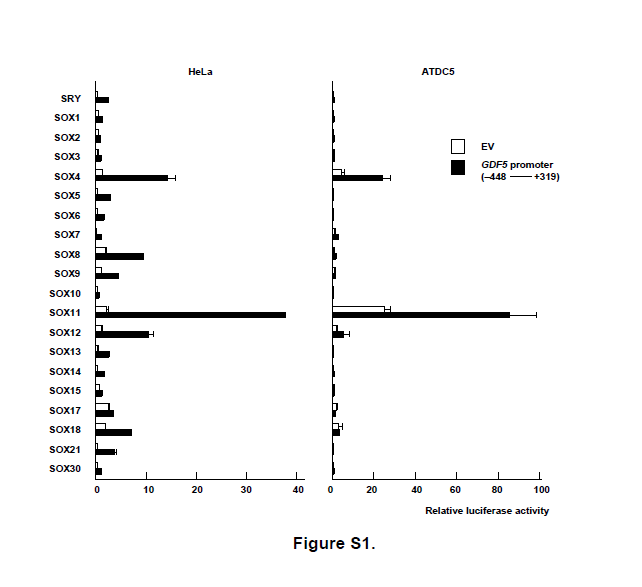

Supplement: Additional file 1: Figure S1 — Screening for candidate SOX genes activating GDF5 promoter. Luciferase assays for the transcriptional activity of the GDF5 promoter by the overexpression of different SOX family molecules in ATDC5 and HeLa cells transfected with GDF5 promoter region (-448/+319) ligated to the luciferase-reporter gene. Data are expressed as means (bars) ± SDs (error bars) of Relative luciferase activity for two assays/construct. EV: empty vector, the vector containing the luciferase transgene but lacking the GDF5 promoter. [file 1471-213X-13-4-S1.doc]

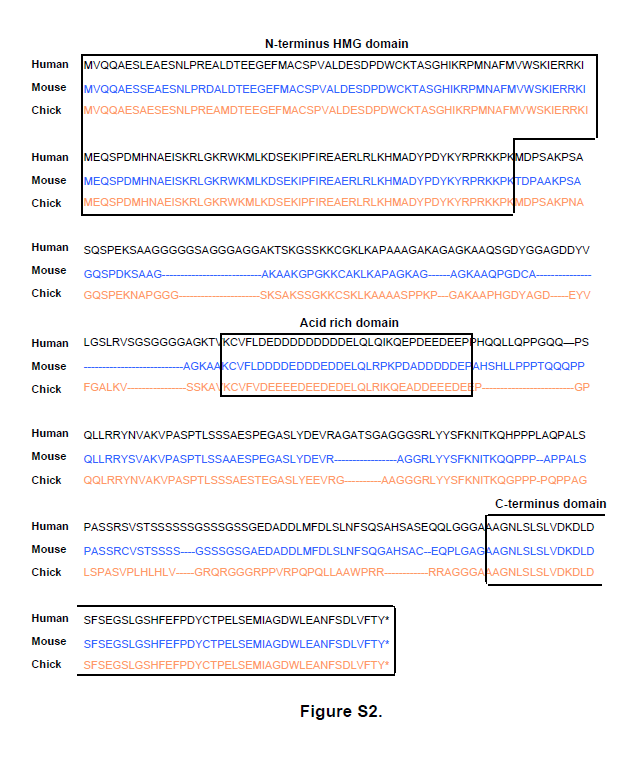

Supplement: Additional file 2: Figure S2 — Conserved domains in the SOX11 protein sequence among humans, mice and chicks. Amino acid sequences coding SOX11 protein is shown and the conserved domains among species are indicated by black boxes. [file 1471-213X-13-4-S2.doc]

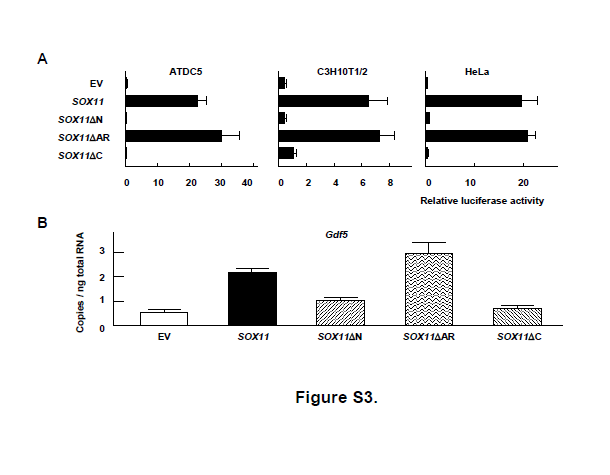

Supplement: Additional file 3: Figure S3 — Identification of functional SOX11 domains which are required for GDF5 expression. (A) Luciferase assays for the transcriptional activity of the reporter driven by the GDF5 (-448/+319) promoter, in the context of co-transfection with deletion mutants that lack N-terminus domain (Rx-ΔN), Acid-rich domain (Rx-ΔAR), or C-terminus domain (Rx-ΔC) in ATDC5, C3H10T1/2 and HeLa cells. Data are expressed as means (bars) ± SDs (error bars) of Relative Luciferase activity for four assays/construct. (B) Endogenous Gdf5 mRNA expressions in ATDC5 cells retrovirally transfected with the deletion mutants of SOX11. mRNA levels were determined by quantitative RT-PCR and expressed as means (bars) ± SDs (error bars) for 4 wells/construct. [file 1471-213X-13-4-S3.doc]

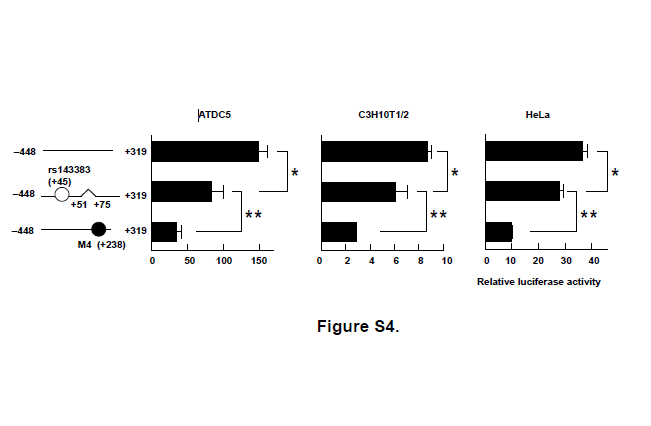

Supplement: Additional file 4: Figure S4 — Luciferase assay for deletion of SOX family binding site near the rs143383. Data are expressed as means (bars) ± SDs (error bars) of Relative Luciferase activity for four assays/construct. *P < 0.05 vs.–448/+319 promoter. **P < 0.05 vs.–448/+319 promoter lacking the +51/+75 region. [file 1471-213X-13-4-S4.doc]

|                 |   |   |   |   |
|-----------------|---|---|---|---|
| SOX11           | - | + | + | + |
| Normal IgG      | - | - | + | - |
| $\alpha$ -SOX11 | - | - | - | + |

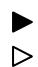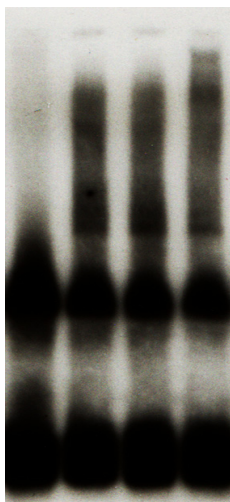

**Figure S5.**

Supplement: Additional file 5: Figure S5 — Electromobility shift assay (EMSA) for specific binding of the GDF5 promoter with human SOX11 protein. The wild-type probe for segment A was used. (Detailed information is shown in Figure 2). An open arrowhead indicates the shifted bands of the SOX11-DNA probe complex, and a solid arrowhead indicates the band supershifted by an antibody to SOX11. The supershift band is absent by a non-immune normal IgG. [file 1471-213X-13-4-S5.pdf]

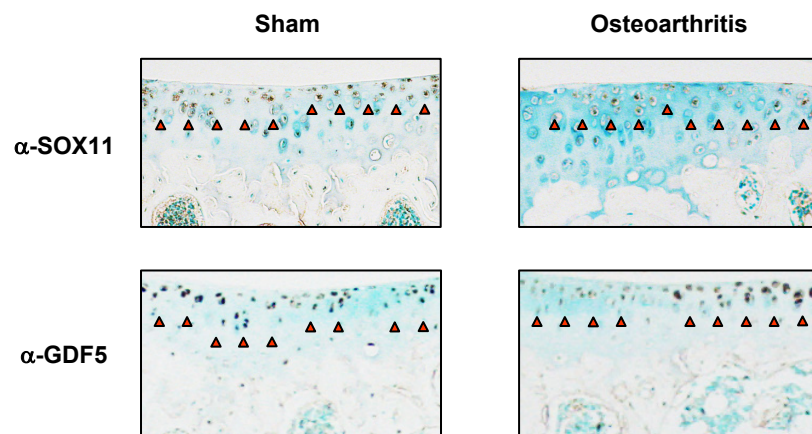

**Figure S7.**

Supplement: Additional file 7: Figure S7 — Immunostaining in the lateral compartment of knee joints in osteoarthritis model of mice. Immunostainings for SOX11 or GDF5 were detected by DAB, which was followed by counterstaining with methyl green. Red arrowheads showed positive signal for SOX11 or GDF5. [file 1471-213X-13-4-S7.pdf]

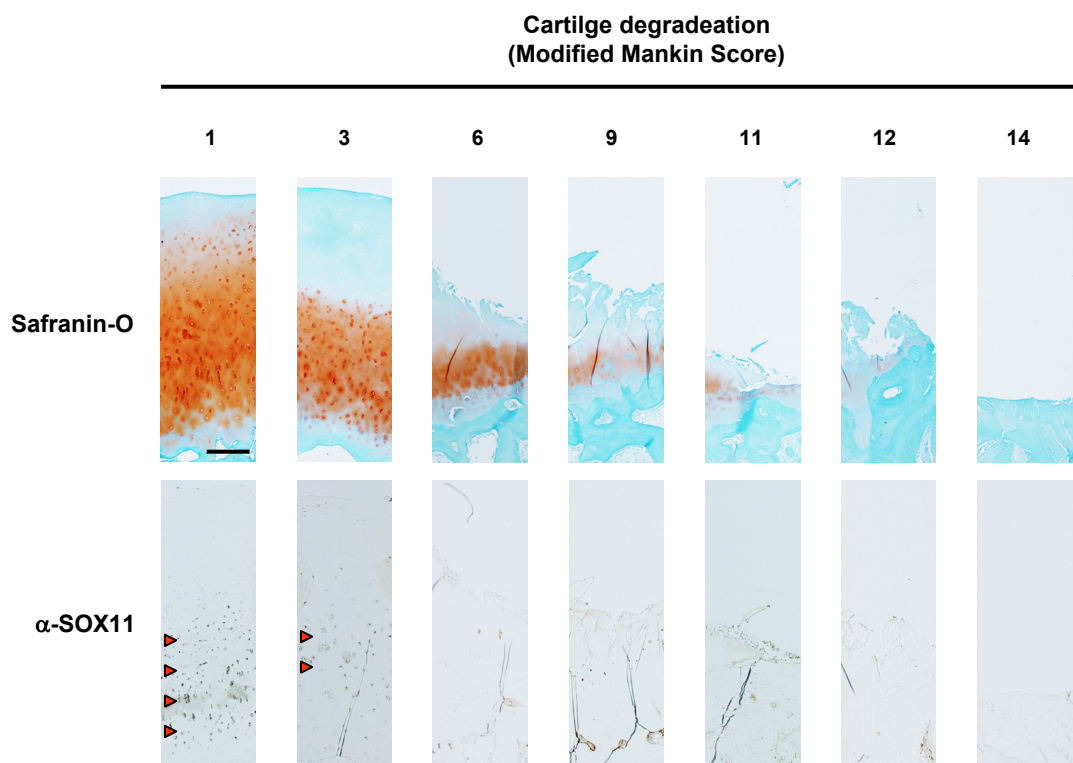

**Figure S8.**

Supplement: Additional file 8: Figure S8 — Decrease of the SOX11 expression during osteoarthritis progression. Safranin-O staining and immunostaining, with an antibody to SOX11, of human knee cartilages. Other representative samples are shown in Figure 5B. Cartilage degradation was evaluated by the modified Mankin scoring system. Red arrowheads indicate positive signal for SOX11. Scale bars, 500μm. [file 1471-213X-13-4-S8.pdf]

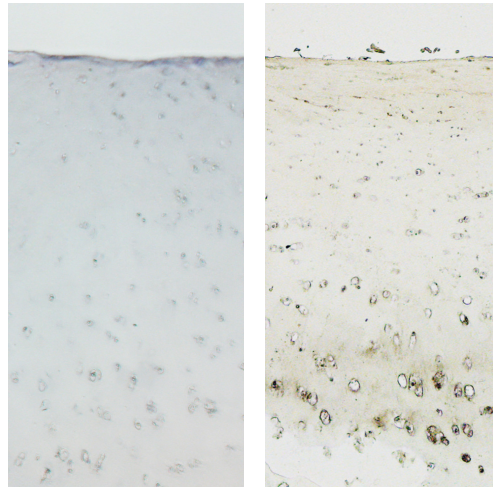

Normal IgG

$\alpha$ -SOX11

**Figure S9.**

Supplement: Additional file 9: Figure S9 — Immunostaining, with an antibody to SOX11 or non-immune normal IgG, of human cartilages. Immunostaining was performed on human knee cartilage with a modified Mankin Score = 2. [file 1471-213X-13-4-S9.pdf]
