# Supplementary material for: SOX11 contributes to the regulation of GDF5 in joint maintenance
Source: BMC Dev Biol. 2013 Jan 29;13:4. doi: 10.1186/1471-213X-13-4 (PMC3760452; doi:10.1186/1471-213X-13-4)
Supplement: Additional file 6: Figure S6 — Immunostaining with an antibody to SOX11 in multiple regions of mouse knee joints. Red arrowheads indicate positive signal for SOX11, while white arrowheads showed scarcity of signal for SOX11. Immunostaining was detected by DAB, which was followed by counterstaining with methyl green. mm: medial meniscus, lm: lateral meniscus, ACL: anterior cruciate ligament. [file 1471-213X-13-4-S6.pdf]

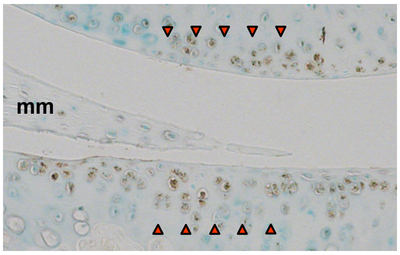

Center of weight bearing area (medial joint)

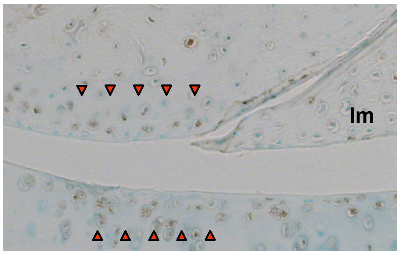

Center of weight bearing area (lateral joint)

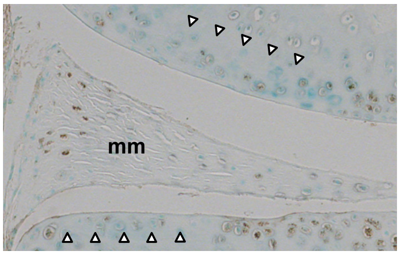

Peripheral weight bearing area

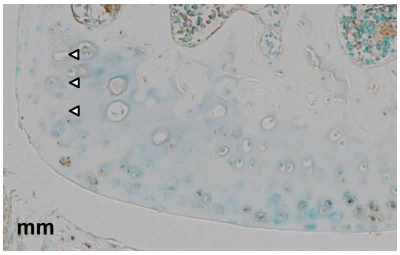

Edge of femoral condyle

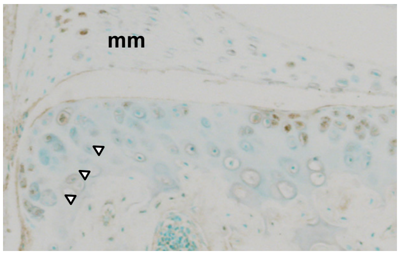

Edge of tibial plateau

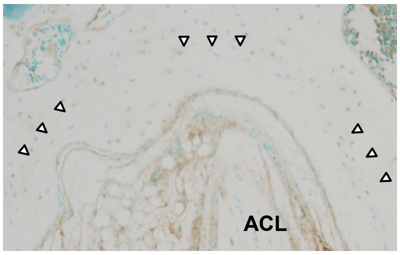

Intercondylar cartilage in femur

Figure S6.
